# GPs and Consultants: Is There Agreement on Patient Management?

**Published:** 1989-08

**Authors:** Michael J. Whitfield, Morag C. Bradley

**Affiliations:** Consultant Senior Lecturer in General Practice General Practice Unit, Department of Epidemiology and Community Medicine, University of Bristol, Canynge Hall, Whiteladies Road, Bristol BS8 2PR Correspondence to Dr. M. J. Whitfield; Research Nurse General Practice Unit, Department of Epidemiology and Community Medicine, University of Bristol, Canynge Hall, Whiteladies Road, Bristol BS8 2PR Correspondence to Dr. M. J. Whitfield

## Abstract

General practitioner attitude questionnaires were sent in May 1987 to 525 general practitioners (GPs) within Avon. A year later a section dealing with the management of clinical situations was sent to 198 Avon consultants, to determine how they would ideally expect a GP to respond to these situations.

The majority of both the GPs and consultants held a common viewpoint, but significant differences were noted between the consultants and GPs in six out of the ten situations. Consultants with more than six months' GP experience had fewer significantly different views than their colleagues with little or no GP experience. GPs and specialists under the age of 45 years also had fewer significant differences in management than older GPs and specialists. The differences seem to reflect the clinical focus and interests of each professional group. We believe vocational training is a contributory factor to the differences and support the General Medical Council's proposal of a broader post registration training for all doctors.


### GPs and Consultants: is there Agreement on Patient Management?

**Michael J. Whitfield, FRCGP**
Consultant Senior Lecturer in General Practice

**Morag C. Bradley, BA**
Research Nurse

General Practice Unit, Department of Epidemiology and Community Medicine,
University of Bristol, Canynge Hall, Whiteladies Road, Bristol BS8 2PR
Correspondence to Dr. M. J. Whitfield

### ABSTRACT
General practitioner attitude questionnaires were sent in May 1987 to 525 general practitioners (GPs) within Avon. A year later a section dealing with the management of clinical situations was sent to 198 Avon consultants, to determine how they would ideally expect a GP to respond to these situations.

The majority of both the GPs and consultants held a common viewpoint, but significant differences were noted between the consultants and GPs in six out of the ten situations. Consultants with more than six months' GP experience had fewer significantly different views than their colleagues with little or no GP experience. GPs and specialists under the age of 45 years also had fewer significant differences in management than older GPs and specialists. The differences seem to reflect the clinical focus and interests of each professional group. We believe vocational training is a contributory factor to the differences and support the General Medical Council's proposal of a broader post registration training for all doctors.

### INTRODUCTION
Patient welfare needs full co-operation between hospital and community sevices. This co-operation in turn depends on collaboration between specialists and general practitioners and has implications not only for high quality patient care but for referral and, ultimately, health authority costs [1].

At present, specialists and general practitioners principally come into contact during the processes of referral and education.

Variations in GPs' rates of referral are still a matter of concern, with no conclusive explanation yet found. Cummins *et al* [2] suggested that doctors have unique 'referral thresholds', and other studies have suggested that diagnostic uncertainty as indicated in Dowie's model of referral [3] is only one aspect of a complex referral decision [4]. Further studies into qualitative aspects such as the 'appropriateness' of GP referrals in terms of how far GPs', consultants' and patients' expectations are met are suggested by Roland [5]. Yet, consultants' involvement in the referral process has been little studied. General practitioners made only fifty-four per cent of the referrals in an outpatient study [3]. Hospital doctors were responsible for the remainder, which included discharged inpatients and referrals from other consultants.

Medical training is mainly hospital based with, more recently, a period in general practice for all students. Since 1982, intending general practitioners have been required to spend two years in hospital training posts and a year as a trainee in general practice. No further general practice experience is required for hospital specialists. The General Medical Council Education Committee, however, identifies a need to continue a broad education beyond full registration [6].

We wanted to know whether GPs and consultants have similar views on clinical management and whether general practice experience affects this.

### SUBJECTS AND METHOD

### Subjects
In May 1987, a general practitioner attitude questionnaire was sent to all 525 general practitioner principals in Avon [7]. Of these, 424 (81%) replied and, excluding practices with restricted list sizes or too few patients in Avon, a final 371 GPs (71%) were entered in this study, which occurred a year later.

To gain consultants' views, the section presenting ten clinical situations and their possible management was sent in July 1988 to 198 clinical consultants employed in four Avon health districts. They were asked how they ideally would expect the GP to answer. Completed questionnaires were received from 166 (84%) of the consultant sample. The distribution amongst the four districts is illustrated in Appendix A and amongst specialties in Appendix B.

There were 250 GPs and 61 consultants under 45 years, 121 GPs and 105 consultants aged 45 years and over. Eighty-four per cent of those consultants with more than 6 months GP experience (25), were in the 'older' consultant sample, but only represented 20% of the total number of consultants 45 years and over. There were 12 female consultants (7.2%); the GP sample included 81 women (21.8%).

### Methods
The GPs were asked to indicate how they would manage 10 clinical situations (scenarios), and the consultants asked how they would ideally expect the GP to manage them, by marking one of four alternatives (Appendix C). The options may be summarised as: (1) Treating symptomatically and/or waiting for the patient to return if necessary; (2) Treating and/or investigating; (3) Treating and/or investigating more extensively; (4) Referring. Scenarios 1, 3, 8, 9, 10 are examples of low risk situations (e.g. scenario 8. Recent onset of colourless, non-offensive vaginal discharge in a menopausal woman. Vaginal examination and speculum examination NAD). Scenarios 2, 4, 5, 6, 7 are higher clinical risk situations because of the patient's age or description of illness (e.g. scenario 4. A 4 year old child with a temperature of 40 °C for 3 days and pain in the head. Physical examination: slight neck stiffness; Kernig dubiously positive; otherwise NAD).

The results were collated and analysed with Kruskall Wallis non-parametric analysis of variance, comparing the answers of: (1) GPs with consultants; (2) GPs with consultants who had more than 6 months general practice experience; (3) GPs with consultants who had no or only locum GP experience; (4) GPs and consultants under 45 years old; (5) GPs and consultants aged 45 years and over.

### RESULTS

The majority of doctors (GPs and consultants) tend to agree on the method of clinical management. Despite this, significant differences emerge. Overall, these differences are because the GPs choose a more conservative management approach, preferring to wait or treat symptomatically (towards option 1) and the consultants expect the GPs to investigate extensively or refer (options 3 or 4).

When comparing GPs and consultants these differences can be seen in scenarios: 2, 5, 6, 7 ($P<0.01$) and 1, 4 ($P<0.05$) (Table 1).

The consultants with more than 6 months' GP experience differ from the GPs in scenarios 5, 6 ($P<0.01$) (Table 2). Those with no or only locum experience differ in scenarios 5, 6, 7 ($P<0.01$) and 2, 4 ($P<0.05$) (Table 3). Therefore, consultants with general practice experience have fewer significant differences to GPs than their colloeagues with no or only locum experience.

GPs and consultants under the age of 45 years differ in scenarios 5, 7 ($P<0.01$) where a higher percentage of GPs are again choosing to wait (towards option 1) and more consultants expect extended investigations or referral (Table 4). This tendency is more extreme in their older colleagues who differ in scenarios 2, 5, 6, 7 ($P<0.01$) (Table 5). Younger GPs and consultants in this study, therefore, had fewer significant differences than older GPs and consultants.

**Table 1**

**Comparison of GPs and Consultants Replies to Senarios 1–10**

| *Scenario* | 1* | | | | 2** | | | | 3 | | | | 4* | | | | 5** | | | |
|---|---|---|---|---|---|---|---|---|---|---|---|---|---|---|---|---|---|---|---|---|
| Option | 1 | 2 | 3 | 4 | 1 | 2 | 3 | 4 | 1 | 2 | 3 | 4 | 1 | 2 | 3 | 4 | 1 | 2 | 3 | 4 |
| GP | 50.1 | 0.8 | 49 | 0 | 57.9 | 11.6 | 28.8 | 1.7 | 18.8 | 45.4 | 34.9 | 0.8 | 1.1 | 19.8 | 1.7 | 77.4 | 27.4 | 58.6 | 13.2 | 0.8 |
| Consultant | 39.5 | 0 | 60.5 | 0 | 44.4 | 13.6 | 36.4 | 5.6 | 21 | 34.6 | 38.3 | 6.2 | 0 | 11.1 | 3.1 | 85.8 | 6.8 | 45.3 | 31.7 | 16.1 |

| *Scenario* | 6** | | | | 7** | | | | 8 | | | | 9 | | | | 10 | | | |
|---|---|---|---|---|---|---|---|---|---|---|---|---|---|---|---|---|---|---|---|---|
| Option | 1 | 2 | 3 | 4 | 1 | 2 | 3 | 4 | 1 | 2 | 3 | 4 | 1 | 2 | 3 | 4 | 1 | 2 | 3 | 4 |
| GP | 10.4 | 54.5 | 34.8 | 0.3 | 10.5 | 75.5 | 2.8 | 11.3 | 11.8 | 9 | 76.7 | 2.5 | 52.3 | 8.2 | 39.2 | 0.3 | 23.1 | 20.9 | 40.4 | 15.6 |
| Consultant | 6.2 | 37 | 56.2 | 0.6 | 2.5 | 62 | 7.4 | 28.2 | 6.1 | 16 | 74.8 | 3.1 | 45 | 10 | 45 | 0 | 5.1 | 37.3 | 44.3 | 13.3 |

n = 365 (GP) 162 (Consultant)
* $P<0.05$
** $P<0.01$
All results are in percentages

**Table 1**

**Comparison of Replies to Scenarios 1–10 between GPs and Consultants with more than 6 months GP Experience**

| *Scenario* | 1 | | | | 2 | | | | 3 | | | | 4 | | | | 5** | | | |
|---|---|---|---|---|---|---|---|---|---|---|---|---|---|---|---|---|---|---|---|---|
| Option | 1 | 2 | 3 | 4 | 1 | 2 | 3 | 4 | 1 | 2 | 3 | 4 | 1 | 2 | 3 | 4 | 1 | 2 | 3 | 4 |
| GP | 50.1 | 0.8 | 49 | 0 | 57.9 | 11.6 | 28.8 | 1.7 | 18.8 | 45.4 | 34.9 | 0.8 | 1.1 | 19.8 | 1.7 | 77.4 | 27.4 | 58.6 | 13.2 | 0.8 |
| Consultant | 33.3 | 0 | 63.7 | 0 | 41.7 | 16.7 | 41.7 | 0 | 24 | 32 | 32 | 12 | 0 | 4.2 | 8.3 | 87.5 | 4.2 | 54.2 | 33.3 | 8.3 |

| *Scenario* | 6** | | | | 7 | | | | 8 | | | | 9 | | | | 10 | | | |
|---|---|---|---|---|---|---|---|---|---|---|---|---|---|---|---|---|---|---|---|---|
| Option | 1 | 2 | 3 | 4 | 1 | 2 | 3 | 4 | 1 | 2 | 3 | 4 | 1 | 2 | 3 | 4 | 1 | 2 | 3 | 4 |
| GP | 10.4 | 54.5 | 34.8 | 0.3 | 10.5 | 75.5 | 2.8 | 11.3 | 11.8 | 9 | 76.7 | 2.5 | 52.3 | 8.2 | 39.2 | 0.3 | 23.1 | 20.9 | 40.4 | 15.6 |
| Consultant | 8.3 | 20.8 | 70.8 | 0 | 4 | 76 | 0 | 20 | 4.2 | 8.3 | 79.2 | 8.3 | 38.1 | 14.3 | 47.6 | 0 | 0 | 30.4 | 56.5 | 13 |

n = 365 (GP) 25 (Consultant)
* $P<0.05$
** $P<0.01$
All results are in percentages

**Table 3**

**Comparison of Replies to Senarios 1–10 between GPs and Consultants with no or only Locum GP Experience**

| *Scenario* | 1 | | | | 2* | | | | 3 | | | | 4* | | | | 5** | | | |
|---|---|---|---|---|---|---|---|---|---|---|---|---|---|---|---|---|---|---|---|---|
| Option | 1 | 2 | 3 | 4 | 1 | 2 | 3 | 4 | 1 | 2 | 3 | 4 | 1 | 2 | 3 | 4 | 1 | 2 | 3 | 4 |
| GP | 50.1 | 0.8 | 49 | 0 | 57.9 | 11.6 | 28.8 | 1.7 | 18.8 | 45.4 | 34.9 | 0.8 | 1.1 | 19.8 | 1.7 | 77.4 | 27.4 | 58.6 | 13.2 | 0.8 |
| Consultant | 41.4 | 0 | 58.6 | 0 | 45.9 | 15.3 | 32.4 | 6.3 | 18 9 | 37.8 | 39.6 | 3.6 | 0 | 10.8 | 1.8 | 87.4 | 7.3 | 42.7 | 32.7 | 17.3 |

| *Scenario* | 6** | | | | 7** | | | | 8 | | | | 9 | | | | 10 | | | |
|---|---|---|---|---|---|---|---|---|---|---|---|---|---|---|---|---|---|---|---|---|
| Option | 1 | 2 | 3 | 4 | 1 | 2 | 3 | 4 | 1 | 2 | 3 | 4 | 1 | 2 | 3 | 4 | 1 | 2 | 3 | 4 |
| GP | 10.4 | 54.5 | 34.8 | 0.3 | 10.5 | 75.5 | 2.8 | 11.3 | 11.8 | 9 | 76.7 | 2.5 | 52.3 | 8.2 | 39.2 | 0.3 | 23.1 | 20.9 | 40.4 | 15.6 |
| Consultant | 4.5 | 41.4 | 53.2 | 0.9 | 2.7 | 55.9 | 9.9 | 31.5 | 5.4 | 17 | 75 | 2.7 | 43.8 | 10.7 | 45.5 | 0 | 6.5 | 37 | 40.7 | 15.7 |

n = 365 (GP) 114 (Consultant)
* $P<0.05$
** $P<0.01$
All results are in percentages

**Table 4**

**Comparison of Replies to Scenarios 1–10 between GPs and Consultants aged 45 Years and Over**

| *Scenario* | 1 | | | | 2** | | | | 3 | | | | 4 | | | | 5** | | | |
|---|---|---|---|---|---|---|---|---|---|---|---|---|---|---|---|---|---|---|---|---|
| Option | 1 | 2 | 3 | 4 | 1 | 2 | 3 | 4 | 1 | 2 | 3 | 4 | 1 | 2 | 3 | 4 | 1 | 2 | 3 | 4 |
| GP | 37.3 | 0 | 62.7 | 0 | 62.1 | 9.5 | 25.9 | 2.6 | 15.8 | 54.4 | 29.8 | 0 | 0.8 | 20.3 | 2.5 | 76.3 | 22.5 | 60 | 15.8 | 1.7 |
| Consultant | 33.3 | 0 | 66.7 | 0 | 38.2 | 14.7 | 41.2 | 5.9 | 22.8 | 35.6 | 36.6 | 5 | 0 | 10.8 | 4.9 | 84.3 | 7.8 | 45.1 | 33.3 | 13.7 |

| *Scenaria* | 6** | | | | 7** | | | | 8 | | | | 9 | | | | 10 | | | |
|---|---|---|---|---|---|---|---|---|---|---|---|---|---|---|---|---|---|---|---|---|
| Option | 1 | 2 | 3 | 4 | 1 | 2 | 3 | 4 | 1 | 2 | 3 | 4 | 1 | 2 | 3 | 4 | 1 | 2 | 3 | 4 |
| GP | 10 | 53.3 | 36.7 | 0 | 12.5 | 73.3 | 5 | 9.2 | 11.6 | 6.6 | 77.7 | 4.1 | 44.6 | 7.4 | 47.9 | 0 | 25.4 | 18.6 | 39.8 | 16.1 |
| Consultant | 5.9 | 32.4 | 60.8 | 1 | 2.9 | 58.8 | 8.8 | 29.4 | 4.9 | 18.4 | 72.8 | 3.9 | 42 | 10 | 48 | 0 | 5.1 | 40.8 | 42.9 | 11.2 |

n = 121 (GP) 103 (Consultant)
* $P < 0.05$
** $P < 0.01$
All results are in percentages

**Table 5**

**Comparison of Replies to Scenarios 1–10 Between GPs and Consultants Younger than 45 Years**

| *Scenario* | 1 | | | | 2 | | | | 3 | | | | 4 | | | | 5** | | | |
|---|---|---|---|---|---|---|---|---|---|---|---|---|---|---|---|---|---|---|---|---|
| Option | 1 | 2 | 3 | 4 | 1 | 2 | 3 | 4 | 1 | 2 | 3 | 4 | 1 | 2 | 3 | 4 | 1 | 2 | 3 | 4 |
| GP | 56.3 | 1.2 | 42.5 | 0 | 55.9 | 12.6 | 30.3 | 1.3 | 20.2 | 41.3 | 37.2 | 1.2 | 1.3 | 19.6 | 1.3 | 77.9 | 29.8 | 58 | 11.8 | 0.4 |
| Consultant | 49.2 | 0 | 50.8 | 0 | 55.9 | 10.2 | 28.8 | 5.1 | 18.3 | 33.3 | 40 | 8.3 | 0 | 11.9 | 0 | 88.1 | 5.2 | 44.8 | 29.3 | 20.7 |

| *Scenario* | 6 | | | | 7** | | | | 8 | | | | 9 | | | | 10 | | | |
|---|---|---|---|---|---|---|---|---|---|---|---|---|---|---|---|---|---|---|---|---|
| Option | 1 | 2 | 3 | 4 | 1 | 2 | 3 | 4 | 1 | 2 | 3 | 4 | 1 | 2 | 3 | 4 | 1 | 2 | 3 | 4 |
| GP | 10.6 | 55.1 | 33.9 | 0.4 | 9.5 | 76.5 | 1.6 | 12.3 | 11.9 | 10.2 | 76.2 | 1.6 | 56.1 | 8.6 | 34.8 | 0.4 | 22 | 22 | 40.7 | 15.4 |
| Consultant | 6.8 | 45.8 | 47.5 | 0 | 1.7 | 66.7 | 5 | 26.7 | 8.5 | 11.9 | 78 | 1.7 | 49.2 | 10.2 | 40.7 | 0 | 5.1 | 32.2 | 47.5 | 15.3 |

n = 245 (GP) 60 (Consultant)
* $P < 0.05$
** $P < 0.01$
All results are in percentages

### DISCUSSION

The interest of this study lies in the similarities or otherwise of a group of GPs and consultants. It is reassuring to find that the main body of GPs and consultants agree on the chosen method of management. Some of the divergence in replies may be explained by the brevity of the scenarios and the limitation of management options. Yet, significant differences were found in a considerable proportion (6/10) of the scenarios. We feel the recognition of these differences is important; they may reflect a lack of understanding between the two parts of the profession to the detriment of effective patient care and efficient use of resources.

The results showed that consultants differed from the GPs by not expecting them to rely on the patient to return if the symptom persists or treat without extensive investigations. Also, the situations where significant differences were shown between GPs and consultants were usually higher in clinical risk. We therefore feel that some of the differences arose through consultants' hospital based training and associated higher incidence of serious pathology leading them to expect extensive investigatons or referrals. Although the sample is small, the fact that consultants with six months general practice experience, and both GPs and consultants under 45 years old (who usually have had undergraduate GP experience) have fewer significant differences to GPs than their less GP-experienced or older colleagues strengthens this point.

Our study showed a tendency for specialists to be more in accord with GPs when answering questions specific to their specialty. Unfortunately, when broken down into specialties, the sample sizes were too small for statistical analysis, and this tendency needs testing with larger samples. Several of the consultants were understandably apprehensive about answering questions on problems outside their specialty. However, from time to time they may be faced with this situatin and be required to make management decisions. They may then refer to specialist colleagues rather than refer the patient back to the general practitioner. The appropriate management decision in some way depends on the doctor's awareness of the available options (GPs now have statutory vocational hospital experience but few consultants have received postgraduate experience of general practice), and on the co-operation of the parties involved.

As one means, therefore, of improving collaboration between primary and secondary health care, we support the GMC's proposal of a broader training, and advocate a period of at least six months general practice experience as part of a clinical specialist's vocational training. The advantages of postgraduate general practice training for all clinicians would include experience of the incidence and pathology of common diseases, a more holistic and preventive approach to illness and demonstrate the GPs' role in providing follow-up care.

### REFERENCES

1. CROMBIE, D. L., FLEMING, D. M. (1988) General practitioner referrals to hospital: the financial implications of variability. *Health Trends* **20**, 53–56.
2. CUMMINS, R., JARMAN, B., WHITE, P. (1981) Do general practitioners have different 'referral thresholds'? *Br. Med. J.* **282**, 1037–1039.
3. DOWIE, R. (1983) General practitioners and consultants: a study of outpatients referrals. London: King Edward's Hospital Fund for London, 1983.
4. WILKIN, D., SMITH, A. G. (1987) Variation in general practitioners' referral rates to consultants. *J. R. Coll. Gen. Pract.* **37**, 350–353.
5. ROLAND, M. (1988) General practitioner referral rates. Interpretation is difficult. *Br. Med. J.* **297**, 437–438.
6. General Medical Council: Education Committee (1987) Recommendations on the training of specialists. London October 1987.
7. WHITFIELD, M., BUCKS, R. (1988) General practitioners' responsibilities to their patients. *Br. Med. J.* **297**, 398–400.

### APPENDIX A

### The Consultants

| District total consultants | sent | Total questionnaires analysed | % of |
|---|---|---|---|
| Bristol & Weston | 100 | 85 | 51 |
| Southmead | 30 | 27 | 17 |
| Frenchay | 26 | 18 | 11 |
| Bath | 42 | 36 | 21 |
| Totals | 198 | 166 | 100 |

### APPENDIX B

### Specialties of Consultant Population

| Specialties | Number of consultants |
|---|---|
| General Medical[1] | 42 |
| General Surgical[2] | 16 |
| Paediatric | 16 |
| Obstetrics and/or Gynaecology | 14 |
| Orthopaedics/Trauma/Accident and Emergency | 15 |
| E.N.T. | 9 |
| Radiotherapy/Oncology | 5 |
| Geriatrics | 7 |
| Psychiatry | 27 |
| Total | 166 |

[1] Including Nephrology, Venereology, Respiratory, Cardiology, Dermatology, Rheumatology, Neurology and Diabetes.
[2] Including Urology, Cardiothoracic, Plastic Surgery.

### APPENDIX C

### The 10 Questions and Initial Response Options

1. A 45-year-old male smoker with a non-productive cough after a course of antibiotics for a respiratory infection. Examination of respiratory system: NAD
   1 If complaint persists—return in 2 weeks
   2 Full blood count, result determines further action
   3 Full blood count and/or CXR, result determines further action
   4 Referral to Chest Physician

2. A 62-year old male, past history of peptic ulcer, complains of dyspepsia at night, nausea and vomiting for two days. Abdominal examination NAD.
   1 General advice, if complaint persists return in two weeks
   2 Faecal occult blood examination, result determines further action
   3 Faecal occult blood examination and/or Barium Meal and/or endoscopy. Result determines further action.
   4 Referral to consultant for diagnosis.

3. A 54-year-old woman complains of stiff wrists in the mornings. Examination reveals symmetrical swelling of the distal interphalangeal joints of both hands and moderately restricted painful joints of both hands and wrists.
   1 Treatment with analgesics and if complaint persists return in two weeks
   2 Full blood count and Rheumatoid Factor, result determines further action
   3 Full blood count, Rheumatoid factor and/or joint X-rays of both hands, results determine further action
   4 Referral to consultant for rheumatological diagnosis and treatment

4. A 4-year-old child with a temperature of 40°C for 3 days and pain in the head. Physical examination: slight neck stiffness, Kernig dubiously positive, otherwise NAD.
   1 Analgesics, ask the parents to contact GP again if necessary
   2 Analgesics, visit within 24 hours to assess
   3 MSU and full blood count, result determines further action
   4 Refer for paediatric opinion

5. A 32-year-old woman with a past history of ear infections and discharging ears in childhood. For one week has right sided earache and offensive aural discharge. Examination shows the auditory canal to be full of pus. Tympanic membrane not visible.
   1 Antibiotic eardrops and/or systemic antibiotics. If complaint persists to return in 3 days
   2 Aural toilet together with antibiotic eardrops and/or systemic antibiotics. Result determines further action
   3 Antibiotic eardrops and/or systemic antibiotics; return in 3 days for hearing tests and otoscopy and for X-ray. Result determines further action
   4 Referral to ENT surgeon

6. A 72-year-old man feels sick for one week then has five loose motions a day.
   1 Diet, if complaints persist return in one week
   2 Physical examination including rectal examination. Result determines further action
   3 Physical examination including rectal examination, stool culture and/or occult blood examination. Results determine further action
   4 Referral to consultant

7. A 48-year-old man developed a shooting pain in the back whilst at work. For one day the pain has been radiating down the left leg. Examination shows loss of sensation over the shin and medial side of the foot and an absent left ankle reflex.
   1 Analgesics, if complaint persists return in 1 week
   2 Analgesics; bed rest for 4 days; assess situation again after 4 days with a visit
   3 X-ray of lumbar spine; result determines further action
   4 Referral to consultant ortho- or neurosurgeon

8. Recent onset of colourless, non-offensive vaginal discharge in a menopausal woman. Vaginal examination and speculum examination NAD
   1 If complaint persists return in two weeks
   2 Cervical Smear taken, results determine further action
   3 Cervical Smear taken and High Vaginal Swab for culture; result determines further action
   4 Referral to gynaecologist

9. A 13-year-old girl with a sore throat. She has been feverish for 4 days (39.5°C). On examination cervical adenopathy, tonsils ulcerated and purulent. No recent history of throat infections.
   1 Treat with suitable antibiotic
   2 Analgesics only + throat swabs; return if indicated
   3 1 or 2 plus full blood count and Paul Bunnel test; results determine further action
   4 Referral to ENT surgeon

10. A 35-year-old man complains of dysuria. He has had one previous episode earlier in the year. Examination of the urinary sediment shows it to be full of pus cells.
   1 Treatment with suitable antibiotic; urine check after treatment
   2 Above (1) plus examination of external genitalia and rectal examination; results determine further action
   3 Above (1) plus examination of external genitalia, rectal examination and IVP; results determine futher action
   4 Referral to urogenital surgeon

